# Enhancing grasping ability with vibrational guidance for individuals who are blind or partially sighted

**DOI:** 10.1038/s41598-025-21720-0

**Published:** 2025-11-04

**Authors:** Katharina C. Trant, Zeynep Bolluk, Rowan A. Mohamed, Sabine U. König, Peter König

**Affiliations:** 1 Institute of Cognitive Science, University of Osnabrück, 49090 Osnabrück, Germany; 2 Department of Neurophysiology and Pathophysiology, University Medical Center Hamburg-Eppendorf, 20246 Hamburg, Germany

**Keywords:** Blindness, Visual impairment, Assistive technology, Tactile bracelet, Grasping guidance, Sensory augmentation, Navigation, Visual system

## Abstract

This study investigates the effectiveness of a vibrotactile bracelet in supporting goal-directed hand movements and grasping tasks for visually conditioned individuals. The research addresses the challenge of object manipulation without visual feedback, comparing tactile to auditory cues. The primary research question explores whether vibrotactile signals delivered via a wrist-worn device can accurately guide hand movements and improve grasping precision. Forty-one participants with varying degrees of limited vision completed a localization task and a grasping task under tactile and auditory guidance conditions and filled out a questionnaire featuring Likert scale items and open-ended questions. The results revealed a mean localization accuracy of 90.6% for vibrotactile signals, with some confusion between neighboring directions. In the grasping task, auditory feedback elicited quicker response times, yet 15 participants exhibited superior performance with tactile feedback. Factor analysis of participant feedback uncovered three key dimensions: confidence, comfort, and learning. Qualitative analysis underscored the need for customizable vibration intensity, enhanced precision, and ergonomic design. Findings suggest that vibrotactile feedback, while slightly slower than auditory cues, is a promising, alternative for supporting grasping without interfering with auditory awareness. Improvements in device design and personalized calibration could enhance its practical application, contributing to greater independence for visually conditioned individuals.

## Introduction

Blindness affects millions of individuals worldwide and presents numerous challenges in daily life^1^. For people with limited vision, activities fundamental to everyday tasks, such as eating, dressing, and manipulating objects, become more complex due to the absence of visual guidance and adjustment of movements. This constraint inherently limits their capacity to fully participate in activities that sighted individuals often take for granted^2^. Recent advancements in assistive Quality-of-life technology, such as obstacle detection devices for the body and the white cane, as well as audio-based navigation apps, have empowered many people with limited vision to achieve greater independence^2,3^. Ultimately, by prioritizing accessibility and innovative solutions, society can significantly enhance the autonomy and quality of life for those with visual conditions.

Especially navigating everyday tasks, like grocery shopping, identifying food, and grasping desired objects, can be particularly challenging for people with limited vision due to their reliance on non-visual senses. The grasping movements representing a central difficulty as vision is the primary sensory system for spatial tasks like grasping^4^. People with limited vision must rely on alternative senses such as touch, hearing, proprioception, and spatial memory to compensate the lack of visual input^5–9^. So, everyday actions are more complex, which highlights the need for accessible design and assistive tools.

Consequently, individuals with visual conditions, despite possessing the physical capacity for grasping, they face significant challenges due to lacking this essential sensory information^4,10^. The developmental foundations of tactile and multimodal-guided reaching in visually conditioned individuals are complex and not yet fully understood. Work by Gori et al.^11^, observed significant differences in spatial perception and sensorimotor responses between sighted and congenitally blind infants when engaging in tactile- and multimodal-guided reaching tasks. These findings strongly suggest that visual experience plays a crucial role in the development of multisensory integration and spatial calibration during early reaching behavior. However, the extent and implications of these developmental differences remain an open question, as compensatory mechanisms and neuroplasticity may allow for alternative strategies in the absence of vision. This developmental perspective underscores the importance of recognizing the diversity of sensorimotor profiles among individuals with visual conditions and the need for tailored assistive technologies that account for such variability in perceptual and motor strategies. In this light, our study does not aim to resolve these developmental questions rather acknowledges the complexity of the field. For us it suffices to note that the relationship between sensory experience and motor behavior is not clear-cut, and we handle our data within this broader context.

In recent years, advancements in assistive technologies have revolutionized the ability of individuals with visual conditions to navigate their environment, localize objects and complete tasks independently. Several assistive technologies have been developed to enhance the autonomy and well-being of individuals with visual conditions^12–14^. These assistive technologies leverage alternative sensory modalities, such as auditory^15–17^ or haptic^18,19^ feedback, to compensate for the lack of visual input.

Auditory substitution is a popular method for compensating for lost vision because it leverages the brain’s cross-modal reorganization, particularly in people with visual conditions, allowing the dorsal visual stream to process auditory spatial information, effectively aiding in tasks that substitute vision with auditory cues^20^. While auditory sensory substitution devices can enhance independence for people with limited vision, they may impede the primary auditory channel, masking essential sounds needed for localization^21,22^. Tactile feedback devices, on the other hand, offer a promising alternative by using sensory augmentation to convey spatial information through touch, avoiding interference with critical auditory inputs^23–25^. By leveraging the sense of touch, these devices enable users to interact with their surroundings more intuitively and safely, enhancing overall situational awareness. Tactile sensory substitution, like auditory substitution, effectively compensates for lost vision by engaging cross-modal reorganization in the brain, particularly in early visually conditioned people, allowing them to process tactile information through visual cortical areas and perform visual-like tasks using tactile cues^18,26^. These developments underscore the remarkable adaptability of the brain and the potential for assistive technologies to significantly enhance the independence and quality of life of those with visual conditions.

Building on the advancements in sensory substitution technologies, researchers have also explored vibrotactile feedback to aid individuals with visual conditions in navigating their surroundings. For example, the feelSpace naviBelt, which is a vibrotactile navigation aid worn around the waist that continuously signals the direction of magnetic north through subtle vibrations, enabling people with visual conditions to maintain orientation and navigate more confidently. It provides an intuitive, hands-free, and attention-sparing sense of direction, enhancing spatial awareness, emotional security, and independence in everyday mobility tasks^9,27,35^. The feelSpace naviBelt has been further investigated, exploring how humans can learn new sensorimotor contingencies through long-term use of the belt, leading to integration of magnetic orientation cues, changes in navigation behavior, and profound alterations in spatial perception^8,28,29^. Its current iteration serves as an assistive device for individuals with visual conditions or blindness without interfering with the auditory sensory channel. Additionally, it can be integrated with a smartphone and a dedicated app, enabling its use as a wearable navigation system^35^. This technology highlights the potential for non-visual, non-auditory sensory feedback to improve autonomy and spatial orientation, enhancing the toolkit available for individuals with visual conditions.

While these studies have provided valuable insights into the potential of vibrotactile feedback for spatial guidance, the applications have been primarily focused on larger-scale navigation rather than the finer aspects of object manipulation, such as reaching and grasping. Such devices are still scarce, but some devices support people with limited vision in some way while grasping. For example, PalmSight^30^ is a device that provides tactile feedback via vibrating motors on the back of the user’s hand, utilizing visual information captured by a camera mounted on the palm. Further, GuideCopter^31^ is a drone-based haptic guidance interface that employs a distinct form of haptic feedback to deliver fine-grained haptic cues and physically direct the user’s hand during hand-object localization tasks in unfamiliar environments. The drone can physically push or pull the user’s hand towards the target object, providing a more direct and intuitive way to guide their movements and interact with the environment.

While these existing devices offer promising ways to support grasping and hand-object localization, they present notable limitations, such as disrupting grasping or lacking practicality for daily use. The placement of tactile feedback on the hand could disrupt the user’s grasping ability. Additionally, a drone-based interface may not be suitable for everyday practical use, which is a critical consideration for individuals with visual conditions, as grasping is a particularly challenging task, and the device’s ease of use in daily life is essential. Moving forward, developing more user-friendly and effective tactile-based solutions will be crucial in overcoming these challenges and ensuring that individuals with visual conditions can navigate both their environments and fine motor tasks with greater ease and confidence.

In light of the limitations of existing tactile and sensory augmentation devices to support hand guidance and grasping, our study investigates an approach that seeks to overcome these challenges by developing a vibrotactile bracelet to assist individuals with visual conditions during object grasping^34^. The tactile bracelet aimed to leave the auditory channel unobstructed while providing precise information via vibrotactile signals on the wrist for hand guidance. By positioning the device on the wrist, the user’s hand remains unencumbered while providing sufficient spatial resolution to distinguish between the four vibration motors corresponding to the up, down, left, and right movement directions. Activating the respective motors guides the user’s hand to the approximate location of the target object, after which the participant can leverage their natural sensorimotor skills to complete the grasping task. This design approach focuses the device’s functionality on the localization and grasping of objects which is particularly challenging for individuals with visual conditions.

Here, based on the previous study in sighted blindfolded participants^32^, we address the question of whether vibrotactile signals applied via a bracelet on the wrist are suitable to support hand movements and grasping in people with limited vision, compared to auditory signals. Thus, we conduct a comprehensive evaluation of a vibrotactile bracelet when used by participants with limited vision and whether it supports localization of target objects and grasping movements. This pilot study explores the device’s potential as a foundation for future assistive technologies. Ongoing research is testing a more autonomous version that replaces the experimenter with an object recognition AI for real-time feedback, marking a key step toward scalable, user-independent systems^33^.

In this study, we explore the effect of conditions (auditory and tactile feedback) as well as task familiarity on grasping performance in people with visual conditions using a vibrotactile bracelet. Specifically, we compare two conditions—auditory and tactile—with the goal of evaluating how participants adapt to the tactile system over time and whether tactile commands can be as good as auditory commands in assisting with grasping movements. Our primary measures of interest for the grasping task are response time (RT) of successful trials as a graded measure of participant engagement and system usability, as well as incorrect/correct responses (accuracy) as a binary measure to see whether participants understood the tactile signal correctly in the localization task. Based in the previous study by Powel et al.^32^, RT continues to reflect learning and adaptation, hence we chose to analyze only successful trials to capture meaningful differences in participant performance in the grasping task.

Specifically in our research, we propose three hypotheses: 1) the **Modality Hypothesis**, which states that participants’ RTs differs significantly between two feedback modalities, the auditory and tactile conditions. This hypothesis tests whether tactile feedback deviates from the auditory feedback; 2) the **Learning Hypothesis**, which states that participants RTs improve significantly over time, suggesting that task familiarity across blocks facilitates learning and enhances performance in both feedback modalities; and 3) the **Position Hypothesis**, which suggests that the spatial position of the target object will significantly influence RTs across both feedback modalities, reflecting the variability of object locations in real-life grasping tasks.

The aim of our study is to investigate whether tactile feedback can support efficient performance comparable to auditory feedback, whether repeated exposure leads to performance improvements over time, and whether object location systematically affects response times across feedback conditions, to provide a viable alternative to the auditory feedback.

## Methods

### Participants

To maximize the recruitment of participants with visual conditions, we were fortunate to leverage the database provided by the company “feelSpace”. We reached out to individuals who were in contact with feelSpace before and had permitted further contact. Moreover, we contacted the ‘Blindenverband Niedersachsen’ (Blind Association of Lower-Saxony) as well as other blind associations close to Osnabrück via email, requesting the possibility of including our recruitment message in their email newsletter to enhance outreach. Afterward, we received several inquiries from smaller organizations. They expressed interest in the possibility of us conducting testing sessions in their respective cities. This interest stemmed from the fact that many visually conditioned people are not at ease with traveling long distances to Osnabrück for participation. Furthermore, we had the opportunity to attend a exhibition focused on people with visual limitations in Hannover alongside feelSpace. During the event, we engaged with attendees on-site and successfully recruited participants spontaneously.

We successfully recruited a total of 42 participants with visual conditions for our study, comprising 21 females and 21 males, this is the official medical categorization^34^. Participants who experienced persistent migraines, headaches, tremors, uncorrectable speech or hearing difficulties, epilepsy, memory impairments, pregnancy, or were under the age of 18 were excluded from the study at the outset. Due to an experimental error, one participant’s data was excluded from the analysis, resulting in a final sample size of 41 usable participants.

Of the remaining 41 participants, 17 were congenitally blind, while 24 experienced acquired blindness later in life. Among the participants, 34 were completely blind, with 20 of them able to perceive light and/or detect changes in lighting conditions. Three participants were 98% visually impaired, all of whom could perceive light and/or lighting changes. Two participants were 95% visually impaired, both of whom also perceived light and/or lighting changes. One participant was 90% visually impaired and was capable of perceiving light and/or changes in lighting, while one participant was 80% visually impaired and did not provide information regarding their ability to perceive light or lighting changes.

### Technical setup

**Fig. 1:**
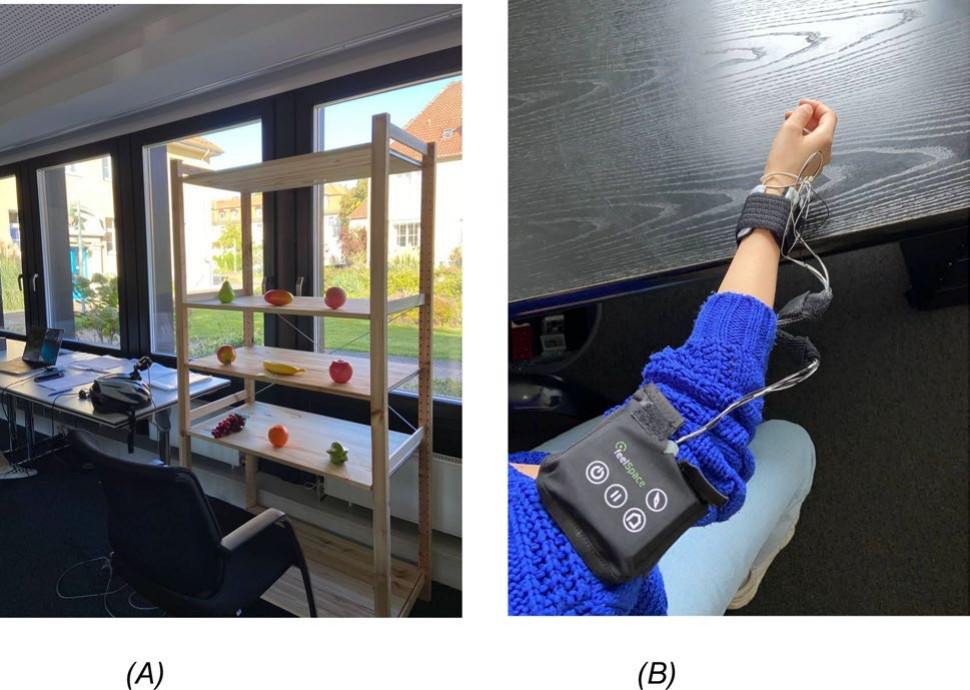
(**A**) Experimental setup: This figure presents the experimental setup, which includes nine plastic fruits on a shelf. (**B**) Vibration bracelet: This figure illustrates the bracelet’s design and how the participants wear it.

The bracelet, shown in Fig. 1B, employed in our study is a prototype derived from the feelSpace naviBelt^9^ and the technical aspects are documented in detail in a previous publication^34^. The bracelet consists of four vibration motors, which were also used for the feelSpace naviBelt, indicating each of the four directions (top, bottom, left, and right), providing vibrotactile input at 0°, 90°, 180°, and 270° around the participant’s wrist, and is worn on the dominant hand. The vibration feedback in our setup was generated using cylindrical eccentric rotating mass (ERM) motors (Precision Microdrives, Model 306-10H). These coreless, precious metal brush DC motors have a 7 mm body diameter and 24.5 mm length, operate at a rated voltage of 3 V, and produce a typical vibration amplitude of 1.84 G at 13,800 rpm with a nominal operating current of 50 mA. The ERM principle produces vibration by rotating an unbalanced mass around the motor shaft, converting the rotational motion into a haptic stimulus suitable for tactile feedback applications. The vibration characteristics can be highly adapted; in this study, the default intensity was set to 100 on a scale from 0 to 100.

In the current version, the bracelet uses a fixed mapping on allocentric coordinates. That is, when a rightward movement is needed and the bracelet is worn on the right arm, the ulna vibration motor is switched on. At this stage of development the tactile information does not adjust to different hand orientations. Further, this is done independent of any pronation or supination of the hand. Consequently, the ulna vibration motor, that in a palm down hand gesture is on the lateral/right side, might point medially when the hand in an extreme supination position.

The motors are attached to a flexible strap that can be adjusted to fit the participants’ wrist sizes. The bracelet was controlled remotely by the experimenter via a laptop, which transmitted commands through a control box attached to the participant’s upper arm. The experimenter only had access to a webcam feed showing the participant’s hands and the target objects on the shelf. Auditory guidance was provided through the laptop’s speakers, where a computer-generated voice repeated the directional command (e.g. “right”, “left”, “up”, “down”, and “grasp”) until the experimenter pressed a button to change the command. Typically, one or two directional commands were followed by a grasping command, which was likewise repeated until the participant grasped and slightly lifted the target object. The experimenter manually terminated each trial upon task completion and documented its success, while the program simultaneously measured and recorded the participant’s response time. The response time includes all hand movement steps, moving in the indicated directions as well as the grasping movement.

In the tactile condition, the same procedure was used, but instead of auditory cues, the bracelet delivered vibrotactile stimuli, the directional vibrations were continuous, lasting as long as the participant needed to move their arm in this direction. The vibration for the grasping command was a pulsation, meaning the upper vibration module would pulsate to give a different sensory sensation than the directional cues. Each pulse was active for 150 ms. This means the signal stayed “on” briefly before switching off again. The total time from the start of one pulse to the start of the next was 500 ms. Since the “on” time was 150 ms, each pulse was followed by 350 ms in the “off” state. Each series contained exactly nine such pulses. At a 500-ms period per pulse, the complete set of pulses lasted 9 × 500 ms = 4500 ms. The time from the start of one pulse series to the start of the next is 5000 ms. This means there was a 500-ms pause after the last pulse before the next series would begin. The pulsation series would repeat as long as the participant needed to grasp the target object and lift it a few centimeters, after which the trial was terminated by the experimenter.

The experimenter only used the arrow keys on the keyboard to indicate the direction command, the “g” button for giving the grasping command, as well as the “s” or “f” button to record if the trial was successful or not. Each button only needed to be pressed once, and the stimuli (auditory and tactile) would persist until another button is pressed.

Participants were seated in front of a three-tiered shelf on which nine plastic fruits were positioned as target objects, each within comfortable grasping distance. The experimental setup is illustrated in Fig. 1A*.*

The camera, an Insta 360 One R with a 1-inch wide-angle lens, is positioned on a standard bicycle helmet to capture a clear view of the region the participant faces. This enabled the experimenter to use this image to provide cues for different directions and the grasping command.

### Procedures

Upon arrival, participants were greeted, briefed about the experiment and task to be completed, and given consent forms and a medical questionnaire. These explanations were given verbally or in audio files, and participants may provide verbal answers and consent if preferred. The participants were informed that they could ask questions at any point and withdraw from the study without any legal implications.

After reviewing, understanding, and signing all necessary documents, participants were guided to a shelf containing artificial fruits, where the experimental tasks would occur. The participants were seated in front of the shelf within an individually adjusted distance considering varying arm’s length. The 9 plastic fruits were placed on the shelf at horizontal and vertical distances of ~ 30 cm and at a depth (measured from the front of the shelf) of about 10 cm. On average the distance of participant to the front of the shelf was about 60 cm and allowed comfortable grasping of the fruits with small movements of the upper body (Fig. 1A). The main experiment involved two conditions: an auditory condition, where verbal directions from a laptop guided hand movement, and a tactile condition, where the bracelet’s vibrations provided directional cues. In consideration of the visually conditioned participants, we chose to begin with the more familiar setup using auditory commands, aiming to enhance their subjective sense of security and support the overall success of the experiment. Participants were instructed to move their arms according to the auditory stimulus. They performed 9 auditory grasping trials across three blocks in this condition (27 in total), with movements closely monitored by a helmet-mounted camera for accuracy and guidance.

After a short break, participants were assisted in putting on the bracelet. To assess their ability to differentiate the signals, each motor vibrated once in a sequence (up, right, down, left), the vibration was active for 500 ms before switching off. This served as a pretest to ensure participants were comfortable wearing the bracelet, to give them the opportunity to familiarize themselves with the vibration sensations, and to verify the bracelet was positioned correctly for each participant, as wrist sizes differ quite a bit. This procedure was repeated until the participants felt comfortable and confident in differentiating the signals from the different directions; on average, participants required three rounds. This also allowed us to adjust the motor positions according to the participants’ needs, ensuring they could differentiate between the signals before starting the actual tactile part of the experiment: the localization task.

Following this, the participants completed the localization task with the tactile bracelet to train themselves with directional vibration signals, ensuring they could accurately identify and respond to tactile cues. After participants completed the localization task, the tactile grasping task would start. Each participant performed 9 grasping trials across three blocks (27 in total) in the tactile condition as before in the auditory condition. After completing the tasks, participants completed a feedback questionnaire to assess their experience and the bracelet’s effectiveness, offering insights into its potential utility for those with visual conditions.

### Localization task

**Fig. 2:**
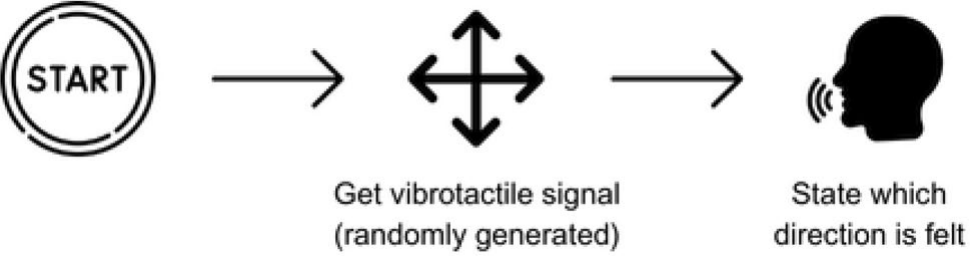
Conceptual representation of the localization task.

We conducted a localization task to test whether participants could accurately identify and respond to tactile cues. It also served as training for using the bracelet in the later grasping task. Once the participants were comfortable identifying the signals and understanding how the bracelet worked, the localization task would begin. The bracelet vibrated once, again for 500 ms, at randomly selected locations, and participants had to loudly indicate the direction of the vibration. This task did not involve hand movements, participants were instructed to sit in a comfortable position and resting their arm either on their leg or the armrest of the chair with the bracelet not touching any of it, to minimize the possibility of malperception of the vibrational signals (Fig. 2). 16 trials comprised one block, and the localization task consisted of three such blocks, summing up to 48 trials in total. This localization task aims to evaluate participants’ ability to identify the location of the tactile stimuli while also serving as a training exercise to familiarize them with the vibration sensation and directional commands.

If participants exhibited discomfort in their responses or if the mean accuracy of the localization task was below 90% after the three blocks, additional trials—typically two supplementary blocks—were administered. Accuracy was then calculated based on performance in the final three blocks. In 14 cases, when the accuracy did not reach 90% even after additional training, we proceeded with the experiment, nonetheless. The participants showed improvement in the task, which we deemed significant progress.

### Grasping task

In the main experiment, containing the auditory as well as the tactile condition, participants grasped and slightly lifted nine plastic fruits placed on the shelf in front of them in a computer-generated random sequence before putting them down again (Fig. 1A). This formed one block of 9 trials, each trial started with giving the first command and lasted until the participant grasped the fruit, we conducted three blocks in each condition summing up to a total of 27 trials per condition and 54 trials in total. The fruits had prototypical forms and were within reach. Participants were instructed to position their hands at the central shelf’s designated starting point (see Fig. 6B) and return to this position after each grasping action. Participants received a demonstration of the operational protocols for auditory and tactile conditions to ensure a comprehensive understanding. Each participant received at least three examples, facilitating familiarity and clarity in task execution before the experimental trials, if this was not enough, they received another round of three demonstrations.

**Fig. 3:**
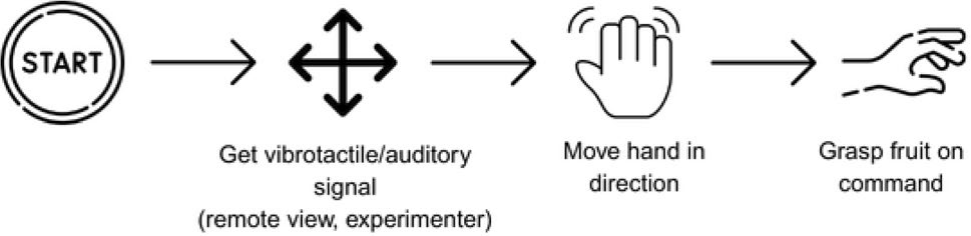
Conceptual representation of the grasping task.

In both conditions, the experimenter observed the scene via the webcam and guided the participants using verbal or tactile commands. Each trial was manually initiated and terminated by the experimenter using a button press. After each trial, the experimenter recorded whether the participant had successfully grasped the target object or if the trial was unsuccessful due to errors made by either the participant or the experimenter (Fig. 3).

In the auditory condition, participants received verbal instructions from a laptop, which included commands like “left”, “right”, “up”, and “down” to guide their hand movements. They were also instructed to perform a grasping movement in response to the word “grasp”. Whereas in the tactile condition, participants received vibrotactile instructions indicating the “left”, “right”, “up”, and “down” directions through continuous vibrations of the respected motor and a short, consecutive vibration from the upper motor to indicate when to grasp. We utilized the laptop’s arrow keys to impart an impulse for the desired direction for both conditions and the “g” key for indicating the grasping command.

### Questionnaire

After completing the experiment, participants were asked to complete a questionnaire of eleven questions, each rated on a five-point Likert scale, where ‘1’ indicates total disagreement and ‘5’ indicates total agreement. At the end of the questionnaire, participants were also given the opportunity to provide additional feedback through open-ended questions. The post-experiment feedback provided valuable insights into the perceived utility of the bracelet among the target demographic. To analyze the responses, we conducted a factor analysis (FA) to identify underlying latent groupings.

To further analyze the qualitative data, we developed a categorization system. Initially, we transcribed the open-ended responses into English. We then created a category system to quantify relevant aspects of the feedback and identify key themes in participants’ experiences. This system was modeled after the framework established by Kaspar et al.^8^. The categorization process was supported by ChatGPT 3.5, which generated preliminary categories based on our data that aligned with Kaspar et al.’s approach. We provided a framework outlining the desired structure of the system and presented our translated statements. The model was then tasked with developing a category system aligned with this framework and organizing our statements accordingly.

We refined the initial ChatGPT generated categories, addressing overlaps and redundancies, ultimately resulting in five Level-1 Categories, each encompassing various Level-2 and Level-3 categories. Definitions were then formulated for each subcategory, and team members independently sorted the participants’ statements into the established categories. Additionally, we presented ChatGPT with our finalized categories and requested that it re-sort our statements according to this updated categorization system. Upon comparison, we found a high degree of agreement in our categorizations, with only minor discrepancies in a few statements, resulting in our final Category System described further below.

## Results

### Localization task

**Fig. 4:**
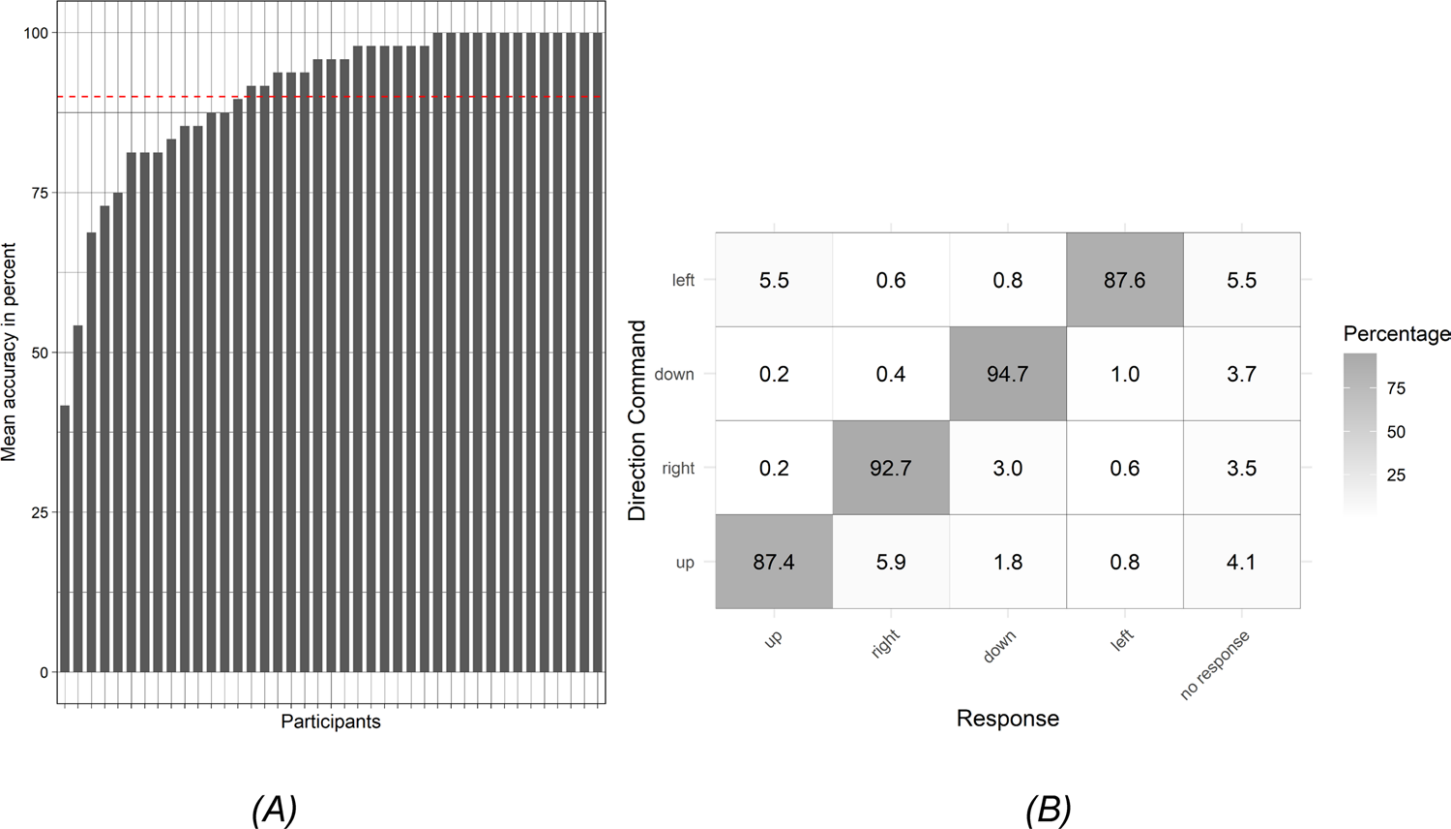
(**A**) Mean accuracy in the localization task by participant. The participants are sorted according to the accuracy reached. The red dashed line represents the mean accuracy across all participants, providing a reference point for assessing individual performance. (**B**) Confusion matrix of vibration signals gives the probability of interpretations for each signal. Correct interpretations are on the main diagonal; confusion of neighboring tactile elements is on the off-diagonal and upper left and lower right corner.

We conducted a localization task to test whether the 41 participants could correctly identify the tactile input from the bracelet. On average, participants achieved an accuracy of 90.6% in localizing the tactile input on the wrist (Fig. 4A). This is slightly above the target accuracy threshold of 90% across the group. However, the spread of accuracy achieved by different participants varied considerably. Out of all the participants, 13 reached a perfect score of 100%, and an additional 15 scored above 90%. Nine participants missed out target accuracy by less than 10%, and five participants had much lower scores. This indicates that the design of the bracelet is working well in many cases but has to be adapted to the individual needs in many other cases.

In Fig. 4B we analyzed how participants interpreted each directional command using the confusion matrix to evaluate the effectiveness of the tactile signals. The confusion matrix summarizes the interpretation of all signals averaged over participants. In the figure, y-axis shows the directional command and x-axis represents the response given by participants. The entries on the main-diagonal, marked with dark grey, shows the correct response, and any off-diagonal entry indicates that a certain vibration element was mistaken as another. We also added cases that participants didn’t give a response to a specific command, which shown in the last column. For example, the direction command “left” was misidentified as “up” in 5.5% of cases, as “right” in 0.6%, and as “down” in 0.8%. It was correctly identified in 87.6% of cases, while no response was given in 5.5% of cases. The accuracy differs only slightly between different directional commands, with down being the most reliable and left and up the most error prone. The errors often occur in neighboring tactile elements, notably up being interpreted as right, right as down, and left as up. Overall, these findings suggest that the separation of tactile elements works well, but the interpretation of neighboring tactile elements has room for improvement.

### Grasping task

**Fig. 5:**
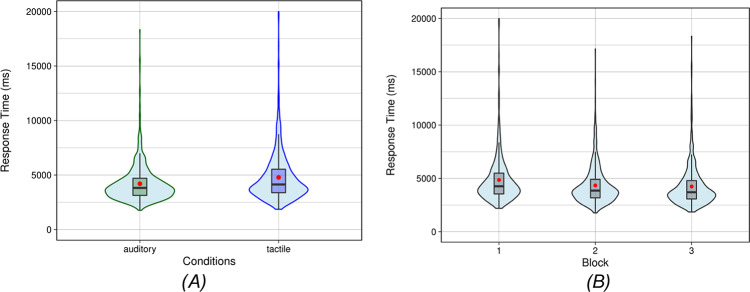
(**A**) Violin plot shows the distribution of response times for all individual grasping movements under auditory and tactile feedback conditions. The plot includes all data points across the entire dataset (not averaged per participant). The box within each violin indicates the interquartile range (IQR), the central line marks the median response time, and the red point indicates the overall mean. (**B**) Violin plot shows the distribution of all individual response times across the three experimental blocks (reflecting task familiarity). Again, the plot includes all data points across the dataset. The boxes represent the IQR, the central line shows the median, and the red point indicates the mean.

To evaluate the effectiveness of tactile feedback compared to auditory feedback in guiding grasping movements, we examined the mean response times over participants across both tactile and auditory conditions from the start of the first signal until grasping the object by only including successful trials. The results showed that the mean trial times for the auditory condition (M = 4198 ms; SD = 1691 ms) were faster than those in the tactile condition (M = 4784 ms; SD = 2255 ms), resulting in an average advantage for the auditory condition of 586 ms (Fig. 5A). Nevertheless, 15 of 41 participants performed faster grasping movements with the tactile guidance than with auditory guidance. Thus, while the auditory condition generally yielded superior results, our findings suggest that tactile feedback also served as an effective alternative for grasping tasks for people with visual conditions. Next, we analyzed the mean response time across the course of the experiment. It is highest in the first block and then decreases in subsequent blocks. Compared to the change from the first to the second block, the change from the second to the third block is much smaller.

To evaluate how feedback modality (tactile and auditory conditions) and task familiarity (blocks 1, 2, and 3) influence grasping performance, we applied a linear mixed model. This model accounted for fixed effects of feedback modality and task familiarity, with random intercepts and slopes to account for individual differences. The model explained 4% of the variance through fixed effects alone (marginal R^2^ = 0.04) and 56% when including random effects (conditional R^2^ = 0.56), suggesting that individual differences contributed substantially to the total explained variance. The analysis revealed a significant main effect of feedback modality (*p* < 0.005), with auditory guidance being faster than tactile guidance. Moreover, task familiarity also had a significant effect on performance. Grasp times improved across blocks, decreasing by 504 ms (*p* < 0.001) in Block 2 and by 624 ms (*p* < 0.001) in Block 3, indicating that participants became faster with practice (Fig. 5B). Although we did not include an interaction term in the final model, our preliminary analyses revealed no significant interaction between feedback modality and task familiarity. This suggests that the effect of task familiarity on grasping performance was independent of the type of feedback provided. This result also aligns with our **Learning Hypothesis** that learning to interpret the signal was equally fast for both conditions. Overall, while auditory feedback proved more effective in improving grasping performance, participants became faster in both conditions as they gained experience, suggesting that tactile feedback remains a viable alternative.

**Fig. 6:**
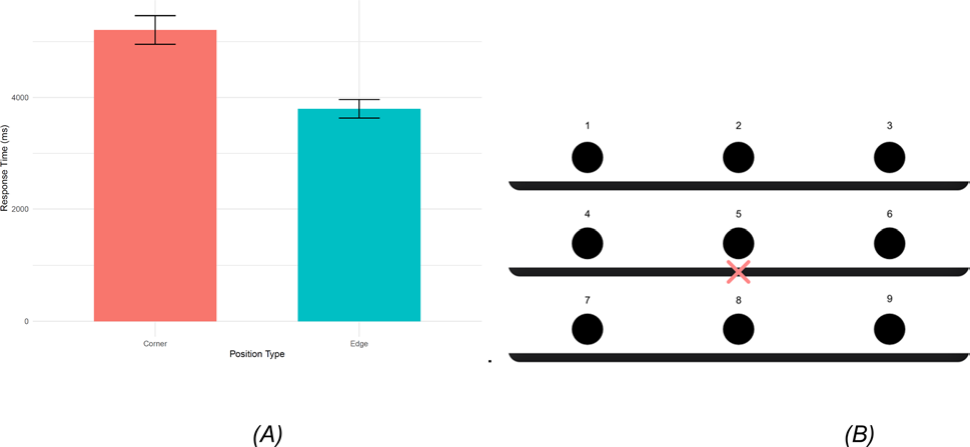
(**A**) Mean response time for corner vs edge positions. The bar graph displays mean response times (in ms) for fruit positions at corners vs. edges. Responses to corner positions were significantly slower than to edge positions, with an average delay of 1410.12 ms. (**B**) Conceptual Experimental Setup: This figure includes fruit position, and the red “X” indicates the starting position for the participant’s hand.

To better understand the role of spatial layout in grasping performance, we conducted an additional analysis based on the **Position Hypothesis**, which proposes that grasping response times are affected by the spatial location of the object, particularly its distance from the central starting position (position 5). The hypothesis assumes that increasing spatial distance increases the variability in RTs. Target positions were grouped into two categories: corner positions (positions 1, 3, 7, and 9), which required three motor commands (two directional and one grasping), and edge positions (positions 2, 4, 6, and 8), which required only two commands (one directional and one grasping). See Fig. 6B for an illustration of the shelf layout. A paired-samples t-test revealed that participants responded significantly faster to edge positions (M = 3797.35 ms, SD = 1056.10) than to corner positions (M = 5207.48 ms, SD = 1640.22), t(40) = 13.52, *p* < 0.001, 95% CI [1199.30, 1620.95], with a mean difference of 1410.12 ms (Fig. 6A). These results suggest that grasping performance is significantly influenced by both the spatial distance from the starting position and the number of required motor commands.

### Questionnaire

We employed a post-experiment questionnaire featuring a quantitative Likert scale and qualitative open-ended questions. The former were analyzed using a Factor Analysis (FA), while the latter was analyzed using a Category System, which allowed quantitative statements.

### Quantitative analysis

To gain insights into user experiences concerning the Likert items of the questionnaire that related to the tactile feedback provided by the bracelet, we conducted a factor analysis employing the Principal Axis Factoring (PAF) method, which aimed to identify underlying constructs influencing participants’ perceptions. A maximum of 1000 iterations was set to ensure convergence of the factor solutions. The resultant factor loadings for each Likert item are displayed in Table 1.

**Table 1:**
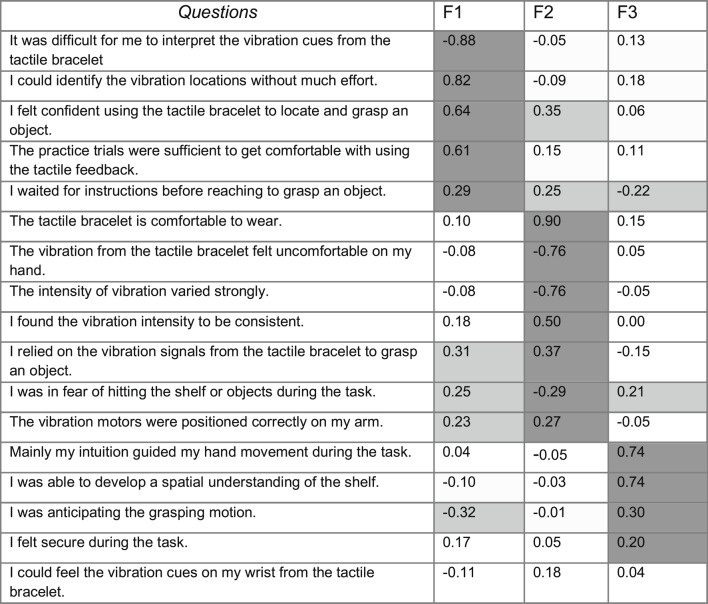
Factor loadings of questions. The questions are listed in decreasing order of loadings on factors 1 to 3. The largest loading is marked in dark grey. Secondary loadings with an absolute value larger than 0.20 are marked in light grey. Any loading below an absolute value of 0.20 is not marked.

The factor loading analysis provides insights into participant responses regarding their experiences with the tactile bracelet. We interpret the first factor as representing “confidence” in the grasping movement. The question of difficulty in interpreting the tactile guidance has a sizeable negative loading. The question of interpretation without large effort has a high positive loading. Further, the explicit question on confidence also has a sizeable positive loading. The second factor centers on “comfort”. The explicit question on comfort is nearly perfectly aligned with this factor. Also, the comfort of perception of the tactile signal has a high load on this factor. However, it must be noted that five questions related to either the first or second factor have a sizable loading on the other factor as well. Thus, the questions do not perfectly separate the two factors of “confidence” and “comfort”. We interpret the third factor as an indicator of the “intuitive” use of the tactile bracelet. Questions with sizable loadings on this factor explicitly mention the intuitive use and the understanding of the spatial setup. Overall, the factor analysis highlights the three dimensions of “confidence”, “comfort”, and “intuition”, where the first two appear related.

As a next step, we compute the average rating of the three factors: confidence, comfort, and intuition. For the factor “confidence”, we obtained a mean rating of 4.1 ± 1.0. For the factor “comfort”, the mean was 4.2, with a standard deviation of 0.9. The mean for the factor “intuition” was 3.6, with a standard deviation of 1.4. These results indicate that all three factors have a rating well above an average score of 3.0 on a Likert scale from 1 to 5. Confidence and comfort of tactile guidance are rated even above 4.0, whereas intuitive use is rated not quite that high.

### Qualitative analysis

In this section, we delve into the qualitative insights gathered from open-ended questions in our questionnaire, aimed at unraveling participants’ perceptions of the tactile bracelet and its associated tasks. In total, 87 sentences were collected from participants, with some providing more than one response. Following thorough organization and preparation, we ended up with 104 statements that serve as the foundation for our qualitative investigation.

We identified five primary categories that encapsulate the diverse feedback, each shedding light on unique aspects of the bracelet: (1) Bracelet Properties, (2) Functionality and Practicality, (3) Bracelet Design, (4) User Experience, and (5) Experimental Setup (see Methods).

A total of 34 statements addressed the first main category: “Bracelet Properties” (Table 2). Of these, six statements concerned the precision of the vibration signal. Two participants expressed the need for increased vibration intensity to improve distinguishability, highlighting the importance of clear directional cues. They suggested making vibration tones more distinct to meet the desire for nuanced feedback. Four participants gave feedback on enhancing precise grasping signals by extending vibrations beyond the upper module for better comprehension and distinguishability. Customization of the bracelet gathered 16 statements and was the focus of the main category. Six participants emphasized the importance of adjustable settings, expressing the need to customize vibration strength, bracelet size, and motor configurations. Additionally, ten participants wished for personalized options regarding vibration strength, size adjustments, and motor strength to have a more tailored experience. Further, 12 statements addressed potential Improvements and Specificity: Four participants emphasized the need for more precise vibration signals, consistently calling for enhancements to make them easier to identify and shorter delays relative to the grasping movements. In addition, they expressed concerns about pseudo-vibrations, aiming to avoid ambiguous or false feedback. The participants also recommended motor enhancement for clearer perception of vibration signals, suggesting smaller and slimmer motors for improved spacing and directional perception. They further suggested expanding the range of vibration possibilities and providing clear indications of directions (up/down, right/left). Overall, the statements in the category of bracelet properties were differentiated and contained many constructive suggestions for future developments.

**Table 2:** Category 1 with all subcategories.

Subcategories of category (1) Bracelet properties		
Level-2	Level-3	Number of statements
Vibration precision (11)	(111) Differentiated signals	2
	(112) Precise grasping signals	4
Customization (12)	(121)Adjustable settings	6
	(122)Personalization	10
Improvements and specificity (13)	(131)Enhanced precision	4
	(132)Avoid pseudo-vibrations	4
	(133)Motor enhancement	4

The second main category of “Functionality and Practicality” gathered a total of 16 statements (Table 3*)*. Six statements concerned the Practical Usage. Three participants identified practical applications for the tactile bracelet at home), suggesting its potential usefulness in locating dropped objects. Meanwhile, three other participants had different opinions about its utility during supermarket shopping, pinpointing specific areas that could be enhanced for a better experience. Five statements related to Safety and Danger Recognition: Five participants expressed their interest in a system capable of recognizing and alerting them to potential dangers in the environment. This emphasizes the practicality of an integrated alert system. Five statements addressed the topic of Object Recognition: Three participants expressed that they wished for advanced object recognition features. Their suggestions were about AI identification capabilities that can recognize various objects. There was also a desire for accurate differentiation of similar items in two participants. The statements in this category demonstrate that the participants would consider the bracelet as the basis for a much larger functionality and as a general-purpose supportive device.

**Table 3:** Category 2 with all subcategories.

Subcategories of category (2) Functionality and practicality		
Level-2	Level-3	Number of statements
Practical usage (21)	(211) Household utility	3
	(212)Supermarket assistance	3
Safety and danger recognition (22)	(221)Obstacle warning	5
Object recognition (23)	(231) AI identification	3
	(232)Product differentiation	2

The third main category of “Bracelet Design” gathered 17 statements (Table 4). Nine statements addressed Bracelet Design: Five participants urged a comfortable, fit, and discreet design, thus recommending a more ergonomic design, enhanced vibration distribution, and adaptable sizing for various wrists. Four participants advocated a discreet and lightweight bracelet design. Four statements addressed Integration and Attachments of the bracelet: Three participants provided diverse preferences for integrating additional components, such as the camera. User preferences regarding camera integration were diverse, from hanging the camera around the neck to placing it outside the face. Hence, customizable options were emphasized for comfort. One participant expected a unified design for seamless compatibility with other technologies and apps for a cohesive user experience. Four further statements addressed Material and Waterproofing: Four participants raised concerns about the bracelet’s material, suggesting alternative materials with water protection properties and improved fastening mechanisms.

**Table 4:** Category 3 with all subcategories.

Subcategories of category (3) Bracelet design		
Level-2	Level-3	Number of statements
Bracelet design (31)	(311)Comfort and fit	5
	(312)Discreet design	4
Integration and attachments (32)	(321) Camera integration	3
	(322) Tech integration	1
Material and waterproofing (33)	(331) Bracelet material	4

The fourth main category “User Experience” contained 28 statements (Table 5). Fifteen of these addressed the adaptation by the user. Generally, nine participants expressed positive sentiments about the quick adaptation of the tactile bracelet. Six users acknowledged the tactile bracelet’s intuitive functionality. However, concerns about imprecision and technical reliability are noted as well. Five statements concerned navigation and orientation: Two improvement suggestions included introducing more variation in direction instructions. Three participants recommended expanding the number of motors for enhanced orientation capabilities. Seven statements addressed Multifunctional Integration: Three participants envisioned a multifunctional device that seamlessly integrates with other technologies, offering expanded functionalities. Four participants proposed features such as facial recognition, reading capabilities, and improved object recognition. Further, one participant emphasized balancing functionality with user and surrounding privacy. They expressed their concerns about unintentional capturing of others via the camera in Concerns about camera usage. Overall, the statements in this main category communicate participants’ positive attitudes towards the tactile bracelet and emphasize potential enhancements.

**Table 5:** Category 4 with all subcategories.

Subcategories of category (4) User experience		
Level-2	Level-3	Number of statements
User adaptation (41)	(411) Quick adaptation	9
	(412) Intuitive use	6
Navigation and orientation (42)	(421) Navigation feedback	2
	(422) Orientation aid	3
Multifunctional integration (43)	(431) Multifunctional usage	3
	(432) Expansion suggestions	4
Privacy concerns (44)	(441) Concerns about camera usage	1

The fifth and final main category relates to the “Experimental Setup”, containing eight statements addressing improvements of the experiment setup and suggestions for further experiments (Table 6). Examples include adjusting the localization setup and announcing signals in the initial round for better attribution. With confidence in our prepared data, we initiate our first analysis by employing Factor Analysis and Principal Component Analysis to extract underlying constructs from numerical responses provided for the questionnaire items. These comments support the positive attitude of participants towards the bracelet and the efforts to evaluate its functionality for visually conditioned participants.

**Table 6:** Category 5 with all subcategories.

Subcategories of category (5) Experimental setup		
Level-2	Level-3	Number of statements
Experiment setup and future directions (51)	(511) How to improve the experiment setup, suggestions for further experiments	8

## Discussion

Our study investigated whether tactile feedback delivered via a bracelet on the wrist could effectively support goal-directed hand movements and grasping in visually conditioned individuals. First, in the localization task, on average, participants achieved a high accuracy in classifying the tactile commands correctly. However, on an individual basis about half of the subjects reached near-perfect performance, while in many other cases, the accuracy was far from optimal. This variability highlights an opportunity for personalized adjustments in signal intensity or patterns to further enhance precision. The bracelet’s ability to support accurate localization underscores its potential as a reliable feedback tool for visually conditioned users.

In the grasping task, our analysis revealed that feedback modality and task familiarity significantly influenced performance in both conditions. The auditory condition that served as a baseline allowed faster grasping, with the difference in time needed being significant but small. Further, on an individual level, 15 of 41 participants performed even better with tactile feedback compared to the auditory baseline. Finally, participants systematically improved as they progressed through the blocks. This highlights the critical role of practice and adaptation in optimizing performance, regardless of the feedback modality, and supports its potential to suit individual preferences.

In addition to the effects of feedback modality and familiarity, spatial layout played a significant role in grasping performance. Our analysis showed that objects located at edge positions (such as the middle left or middle right of the shelf) were grasped significantly faster than those located at corner positions, where an extra directional command was required to guide the participant. Each additional command resulted in an average delay of approximately 1410.12 ms, highlighting the cost of processing and executing multi-step spatial cues. Moreover, this expected delay may show that our shelf design intentionally mimicked real-life contexts, such as a visually conditioned person reaching for an object in a kitchen or storage cabinet, unlike abstract lab setups. The slower response times for corner items suggest that real-world spatial complexity can substantially influence assistive device performance, and systems should be designed with this variability in mind.

The experimental participants gave quantitative feedback. A factor analysis grouped this into three distinct factors: user confidence, comfort, and learning/intuition. All factors were scored above average, and the first two were even above 4 on a scale of 1–5. These results emphasize the potential of tactile input as an effective and user-friendly feature, making it a valuable basis for future device designs.

The qualitative analysis of participants’ feedback identified five key categories: Bracelet Properties, Functionality and Practicality, Bracelet Design, User Experience, and Experimental Setup. Participants highlighted the importance of customizable vibration settings and the potential for real-world applications, such as safety alerts and object recognition. Positive sentiments regarding the bracelet’s comfort and ease of use were expressed, though suggestions for improved precision and technical reliability were noted. The feedback supports the device’s potential as a user-friendly and versatile tool, with clear directions for future improvements.

Learning how to use the tactile bracelet effectively requires time and exercise. In the experimental setup and the context of recording, the training time had to be minimal. Because tactile input to guide grasping direction is a new sensation not commonly present in daily life, a short training phase may have hindered performance. Future research should incorporate extended training or familiarization phases to enhance accuracy and responsiveness.

The insights we gained via the localization task highlight the need for improved vibration patterns to enhance directional cue differentiation. The fixed wrist orientation and static bracelet design also posed issues, suggesting that future designs should dynamically adapt to hand movements and accommodate wrist size variations. Additionally, participants recommended improvements in vibration clarity, customizable settings, and bracelet comfort. Addressing these factors through user-centered design could enhance the device’s practicality and accessibility for daily use by visually conditioned individuals.

Based on our findings, our tactile bracelet fits within the ongoing advancements in assistive technology for individuals with visual conditions. It addresses the unique challenges posed by tactile sensory substitution in object manipulation and grasping. Our results highlight both the promise and limitations of using tactile feedback for hand movement guidance, adding to the discourse on supportive devices for visually conditioned individuals.

Taken together, our findings directly address the hypotheses outlined in our introduction. In line with the **Modality Hypothesis**, we observed a significant difference in response times between the auditory and tactile conditions, confirming that while tactile feedback is not yet as efficient as auditory guidance, it shows strong potential, particularly considering that a substantial number of participants performed better with tactile input. The **Learning Hypothesis** was also supported by the observed improvement in performance across blocks, indicating that familiarity and repetition enhance task execution regardless of feedback modality. Finally, the **Position Hypothesis** was reflected in the systematic variation of response times depending on the spatial location of the object, underscoring the relevance of spatial layout in grasping tasks. These findings validate our initial assumptions and support the broader aim of exploring tactile feedback as a scalable, user-independent alternative to auditory systems in assistive technologies for individuals with visual conditions.

Our results add depth to the discussion on non-visual sensory substitution as an alternative to auditory substitution, particularly for tasks requiring precise control and attention, such as grasping. As the introduction noted, auditory substitution methods are popular due to the brain’s cross-modal plasticity, which allows visually impaired individuals to process spatial information through auditory input^20^. However, these auditory systems may interfere with essential ambient sounds needed for navigation and situational awareness^21^. Our findings suggest that tactile feedback is indeed an effective substitute for object manipulation without blocking auditory input, supporting the idea that tactile feedback can be a valuable, non-intrusive alternative for tasks that require high situational awareness and cannot afford auditory interference^26^.

Here, we aim to extend the use of tactile feedback applications beyond navigation. While the feelSpace naviBelt and similar technologies provide valuable directional cues for navigation, tasks requiring fine motor control, such as grasping, present unique challenges. Our results demonstrated that while participants could effectively interpret general directional cues, specific directional vibrations were often confused, highlighting the need for more refined, easily distinguishable tactile signals. Moreover, our results reinforce prior evidence that tactile feedback systems must be designed to minimize interference with manual task execution. Previous approaches, such as PalmSight and GuideCopter, exemplify these challenges, with the former obstructing grasping ability by mounting the camera on the hand and the latter employing drone-based actuation that physically manipulates the user’s hand, both of which hinder practical daily use^30,31^.

In comparison, our device tries to mitigate these limitations through a helmet-mounted camera, which preserves full hand functionality and does not require some searching movements with the hand and arm to identify target objects as its positioning gives a good view of the participants’ field of view, and by situating the vibration motor on the wrist and the control unit on the upper arm, thereby avoiding intrusive physical manipulation of the hand. These design decisions improve usability and safety for fine motor activities. Nevertheless, despite these advantages, the current helmet-mounted setup remains cumbersome and not ideally suited for everyday use. Rather than presenting a perfected device, this work offers a further attempt toward identifying design strategies that may ultimately lead to a more practical and widely applicable tactile feedback solutions. Further development is required to enhance its wearability and overall practicality for long-term, real-world deployment.

Our study contributes to the understanding of how tactile sensory substitution can be applied in grasping tasks, underscoring the importance of refining tactile signals and considering the diverse sensorimotor backgrounds of individuals with visual conditions, as highlighted by developmental differences observed in earlier work, see Gori et al.^11^. Participant feedback further emphasized the need for greater precision and customizability of the vibrotactile signals, such as clearer directional cues, adjustable vibration strength, and improved motor configurations, reflecting the variability in perceptual strategies and the importance of adaptable assistive technologies. These findings suggest that while tactile feedback holds promise, it may need further development to meet the precision demands of object manipulation tasks.

In a previous study, Powell et al.^32^, investigated using a tactile bracelet in young, sighted, and blindfolded participants. In that study, participants achieved a very high mean accuracy rate of 98% on their initial attempt, the ~ 90% in the present study. Performance differences were also evident in the grasping task. In Powell et al.^32^, auditory guidance facilitated faster trial times by about 500 ms. Similarly, our study found auditory guidance to be more effective than tactile feedback. Comparing the mean trial time differences between the tactile and auditory conditions on an individual level demonstrates a notable fraction of participants with superior performance in the tactile condition. This contrasts with the prior blindfolded study, in which only one participant outperformed in the tactile condition, whereas our study found that 15 of 41 participants had better results with tactile feedback. The reasons for this difference still need to be determined. Candidate explanations include differences in the onset of visual condition, as previously mentioned, 24 participants experienced vision loss later in life, while 17 were congenitally blind, previous experience with other assistive devices, and intrinsic motivation to make a new assistive device work. In any case, the data highlight the promise of tactile feedback in grasping tasks for visually conditioned individuals.

The studies also differed in their approach to participant feedback. Powell et al.^32^ employed Principal Component Analysis to consolidate responses from Likert-scale questions into three principal components: Vibration, Bracelet Overall, and Experiment. Their analysis highlighted high participant confidence and satisfaction, with minor recommendations for refining vibration precision and motor placement. In comparison, our study utilized Factor Analysis, which identified three key factors rated above average: Confidence, Comfort, and Learning. Although there are similarities in the participant feedback, it appears that the visually conditioned and blind persons commented less on the experimental setup and specifics of the vibration but focused more on the user perspective, i.e., on how far the bracelet would allow comfortable and confident use of the device for grasping movements. This interpretation is further supported by the qualitative feedback in the current study, categorized into five distinct factors. That is, the present study participants were willing to evaluate the bracelet from a personal and practical perspective.

We acknowledge that our study design, particularly the use of an experimenter to deliver feedback based on visual input, imposes limitations on the generalizability and autonomy of the system. Ongoing research is already moving toward a more autonomous version, replacing the experimenter with an object recognition AI to provide real-time, user-independent feedback^33^. This advancement represents a significant step toward scalable, user-independent systems that could offer visually conditioned individuals greater autonomy in object localization and grasping. Additionally, we recognize that our controlled laboratory environment, with fixed elements such as shelf dimensions and seating, does not fully capture the complexities of real-world application. Therefore, future studies should aim to test the device in more naturalistic settings to increase ecological validity. Furthermore, variability in participant performance likely reflects individual differences in tactile sensitivity, prior experience, or cognitive strategies. Consequently, personalized calibration of vibration patterns and intensities could improve both accuracy and user satisfaction. Finaly, to give the visually conditioned participants a higher subjective feeling of security, we considered starting with the less alien setup of auditory commands, which would benefit the whole experiment. Future studies could explore starting with a random condition.

Another important limitation to consider involves the potential sources of delay in participants’ motor responses following the tactile cue. One possible explanation is that participants may have needed additional time to process the vibrotactile signal. This is supported by findings from the tactile perception literature. For example, previous studies suggest that the localization and processing of tactile stimuli are influenced by the limb’s configuration and the current postural state^36,37^. In the context of the present study, different movement directions likely resulted in varied wrist or arm postures, which may have affected the timing or ease of interpreting the tactile signal. Furthermore, evidence indicates that voluntary movement can suppress tactile perception^38,39^. This sensorimotor suppression effect may have made it more difficult for participants to perceive or interpret the vibrotactile cue accurately, especially if they were preparing or initiating movement simultaneously. As a result, this could contribute to inconsistent or delayed responses.

The fact that the experimenter simultaneously manages multiple tasks could be another factor contributing to variability in participant response timing. This potential variability likely affected both tactile and auditory conditions similarly. Importantly, the primary focus of our research was to assess whether tactile feedback is as effective as auditory feedback, rather than to precisely identify the sources of motor delays. While these factors were not explicitly controlled in the current study, acknowledging their potential influence highlights an important consideration for future research.

Additionally, we observed 11 individual trials that exceeded 15 s, three of which were in the auditory condition, and 38 trials that lasted longer than 10 s, with 12 of these in the auditory condition. We also acknowledge that some participants may have hesitated, become distracted, or had difficulty moving comfortably during these specific trials due to wearing the helmet with the attached camera and tactile bracelet unit for the tactile condition, or may have found the task challenging due to a combination of these factors.

Importantly, the positive feedback and performance outcomes suggest that the bracelet holds promise as a flexible assistive tool. With training, tactile feedback has the potential to be as effective as auditory input, offering users the freedom to choose the modality that best fits their needs and preferences.

## Conclusion

This study highlights the promising potential of vibrotactile feedback as a robust and accessible tool for supporting goal-directed hand movements and object grasping in visually conditioned individuals. By preserving the auditory channel, the tactile bracelet offers a unique advantage, enabling users to maintain awareness of their auditory surroundings while receiving precise directional cues. Despite challenges such as slower response times and variability in performance, the results demonstrate that tactile feedback systems can effectively complement natural sensorimotor skills and adapt to individual user needs. Moreover, the device was designed to require as minimal training as possible, making it suitable for everyday use and easily integrated into the lives of visually conditioned individuals, ultimately enhancing their independence and quality of life. Overall, the demonstration and training trials typically required less than 10 min, and even when considering the entire tactile experiment as a single extended training session, the total duration per participant was approximately 30 min—an acceptable timeframe for a one-time training procedure.

Our study contributes to the growing body of research on assistive technologies by exploring the application of vibrotactile feedback in fine motor tasks, such as grasping. While previous studies have primarily focused on auditory or large-scale navigational aids, this research extends tactile feedback to more precise tasks, offering a wearable, more practical vibrotactile bracelet as a sensory substitution tool. Such devices could ultimately enhance independence and task-specific capabilities for visually conditioned individuals, addressing the crucial gap in assistive technologies for fine motor tasks in addition to broader navigation support.

## Data Availability

The data presented in this study are openly available at (https://github.com/lingRowan/Shopping-Bracelet-for-Blind-People).
